# Deceleration and acceleration capacities of heart rate associated with heart failure with high discriminating performance

**DOI:** 10.1038/srep23617

**Published:** 2016-03-23

**Authors:** Wei Hu, Xian Jin, Peng Zhang, Qiang Yu, Guizhi Yin, Yi Lu, Hongbing Xiao, Yueguang Chen, Dadong Zhang

**Affiliations:** 1 Department of Cardiology, the Center Hospital of Minhang District, 170 Xinsong Road, Minhang District, Shanghai 201199, China

## Abstract

Accurate measurements of autonomic nerve regulation in heart failure (HF) were unresolved. The discriminating performance of deceleration and acceleration capacities of heart rate in HF was evaluated in 130 HF patients and 212 controls. Acceleration capacity and deceleration capacity were independent risk factors for HF in males, evaluated by multiple logistic regression analysis, with odds ratios (ORs) of 5.94 and 0.13, respectively. Acceleration capacity was also an independent risk factor for HF in females, with an OR of 8.58. Deceleration capacity was the best cardiac electrophysiological index to classify HF in males, with an area under the receiver operating characteristic curve (AUC) of 0.88. Deceleration capacity was the best classification factor of HF in females with an AUC of 0.97, significantly higher than even left ventricular ejection fraction (LVEF). Acceleration capacity also showed high performance in classifying HF in males (0.84) and females (0.92). The cut-off values of deceleration capacity for HF classification in males and females were 4.55 ms and 4.85 ms, respectively. The cut-off values of acceleration capacity for HF classification in males and females were −6.15 ms and −5.75 ms, respectively. Our study illustrates the role of acceleration and deceleration capacity measurements in the neuro-pathophysiology of HF.

Heart failure (HF) refers to structural or functional impairment in the ventricular filling or ejection of blood, which may lead to fluid retention, pulmonary congestion, peripheral edema, and a complex clinical syndrome^1^. HF may be caused by disorders of the myocardium, heart valves, pericardium, and endocardium^2^. Hypertension, chronic obstructive pulmonary disease, and certain metabolic abnormalities are also common etiological factors of HF^3^. HF is a major challenge to public health; in the United States, there are as many as 650,000 new cases diagnosed annually, and this rate has remained stable over the past several decades^4^. The absolute mortality rate for HF is estimated to be ~50% within 5 years of diagnosis^1^,^5^.

The physiological activity of the heart is controlled and modulated by the parasympathetic and sympathetic nervous systems^6^,^7^. The sympathetic nervous system has a wide variety of cardiovascular actions, including heart rate acceleration, increased cardiac contractility, reduction of venous capacitance, and constriction of resistance vessels^7^,^8^. The cardiovascular effects of the parasympathetic nervous system (vagus nerve) include heart rate reduction by inhibiting the sympathetic nervous system and by direct hyperpolarization of sinus nodal cells^6^,^9^. Disorders in parasympathetic and sympathetic nervous systems of the heart may coexist with serious heart consequences, including HF^6^,^9^. Dysregulation of cardiac adrenergic receptor signaling and transduction will influence cardiac inotropy and is a key feature in HF progression^7^,^10^. In contrast, the pathophysiological roles of normal and disordered parasympathetic innervation in heart are not as well understood^6^,^7^,^8^,^9^,^10^,^11^.

The real-time roles of the parasympathetic and sympathetic nervous systems on the heart are difficult to monitor. However, increasing numbers of studies have shown that these roles are reflected in cardiac electrophysiology^6^,^7^,^8^,^9^,^10^,^11^. Heart rate variability (HRV) is the physiological phenomenon of variation in the interval between heart beats, which can be measured and calculated from a continuous electrocardiograph record^12^. In recent decades, time and frequency domain measures of HRV have been considered to represent promising markers of a significant relationship among autonomic nervous system activity, HF, and cardiovascular mortality^12^,^13^. Although evidence for an association between a propensity for lethal arrhythmias and signs of increased sympathetic or reduced vagal activity is abundant, the significance of the many different HRV indexes is more complex than generally appreciated, and there is potential for incorrect conclusions and for excessive or unfounded extrapolations^12^.

In 2006, Baver *et al.* established an approach to distinguish between vagal and sympathetic nervous system roles that affect cardiac electrophysiology using a signal processing algorithm to separately characterize the deceleration and acceleration capacities of the heart rate^14^. The deceleration and acceleration capacities of heart rate were quantified by assessing 24-h ambulatory electrocardiogram recordings^14^,^15^. The authors found that decreased heart rate deceleration capacity was a powerful predictor of mortality after myocardial infarction, better than left ventricular ejection fraction (LVEF), conventional measures of HRV, and the combination of the two^14^. Their report advanced cardiac electrophysiological analysis and provided a new approach to quantify the effects of the vagal and sympathetic nervous systems on heart physiology.

Although elevated sympathetic activity is associated with an adverse prognosis, and a high level of parasympathetic activation confers cardioprotection^6^, a multitude of unknown questions still need to be answered. The parasympathetic actions on the heart are mediated not only by cardiac muscarinic receptor stimulation but also by many known and unknown mechanisms^6^. The role of the vagal nerve in heart biological activity has only recently been investigated in human subjects with HF. Thus, we should consider that any novel approach might advance our knowledge of cardiac nerve electrophysiology. In this report, the significance of acceleration capacity and deceleration in HF was evaluated comprehensively by receiver operating characteristic (ROC) and multiple logistic regression analysis together with echocardiographic and HRV indexes under strict statistical quality control. Our data provide new insights into how parasympathetic and sympathetic activation affects HF.

## Methods

### Participants

Outpatients and inpatients who visited our Cardiology Department from February 2012 to April 2014 were enrolled in the study. HF was diagnosed by disease history, symptoms, and signs, in combination with chest X-ray, electrocardiography, and transthoracic echocardiography results according to the Report of the American College of Cardiology Foundation/American Heart Association Task Force on Practice Guidelines (2013 ACCF/AHA Guideline for the Management of Heart Failure)^1^. The HF classification recommended by the American College of Cardiology Foundation and American Heart Association was adopted for diagnosis of HF stage: stage A, at high risk for HF but without structural heart disease or symptoms of HF, stage B, structural heart disease but without signs or symptoms of HF, stage C, structural heart disease with prior or current symptoms of HF, and stage D, refractory HF requiring specialized interventions^1^. Subjects who visited our department without HF and in whom heart disease was excluded were recruited as controls. The exclusion criteria were a history of atrial fibrillation, atrial flutter, sick sinus syndrome, atrioventricular block, implantation of a pacemaker, and renal insufficiency.

The review board of the Center Hospital of Minhang District approved this protocol in accordance with the amended Declaration of Helsinki. Written informed consent regarding the procedures and medical data to be used was obtained from all patients according to the guidelines of the Chinese National Ethics Regulation Committee.

### Deceleration capacity and acceleration capacity calculations

To calculate the deceleration capacity and acceleration capacity of the patients and controls, original electrocardiogram information for all participants was obtained from 24-h ambulatory electrocardiogram monitoring using a Holter monitoring DigiTrak XT System (Philips, Best, the Netherlands).

The heart rate deceleration and acceleration capacities were calculated according to Baver^14^. Briefly, in Step 1, heartbeat intervals shorter than the preceding interval were defined as accelerating anchors, and heartbeat intervals longer than the preceding interval were defined as decelerating anchors. RR (R, the peak of the QRS complex of the electrocardiogram wave) interval prolongations (or shortenings for acceleration capacity computation) of more than 5% were excluded to avoid artifacts errors^14^,^15^. In Step 2, segments of interval data around the decelerating and accelerating anchors were selected. All segments were chosen according to the lowest frequency to be visualized^16^. In Step 3, all of the above cardiac electrical segments were aligned at the decelerating and accelerating anchors. In Step 4, signal averaging, the phase-rectified signal averaging signal X(i) was obtained by averaging the signals within the aligned cardiac electrical segments. Finally, in Step 5, the acceleration and deceleration capacities were quantified using the formula: DC (AC) = [X(0) + X(1)−X(−1)–X(−2)]/4.

### HRV measure and analysis

All HRV indexes were calculated from 24-h ambulatory electrocardiograms that were recorded under fairly similar conditions and in a fairly similar environment. Abnormal RR intervals, defined as RR intervals that change by more than 20% from the previous RR interval, such as premature atrial contraction (PAC) or premature ventricular contraction (PVC), atrial fibrillation, ventricular tachycardia and etc, were removed from the RR interval series^12^. The exclusion rates of abnormal RR intervals ranged from 5.2%–10.1% and 0.5%–4.1% in HF patients and controls respectively. Low frequency trends were detrended by removing a linear least-squares-fit from the RR interval series. Signal stationarity and regular sampling were accomplished using cubic spline and linear interpolation^12^. The default value for the resampling rate is 3 Hz. Statistical time-domain measures were calculated directly from the NN interval series. The frequency domain measures of HRV were performed by non-parametric method using fast Fourier transform (FFT).

The following parameters related to HRV were determined according to the Task Force of the European Society of Cardiology and the North American Society of Pacing Electrophysiology recommendation^12^: indexes of frequency-domain methods including high frequency (hF), from 0.15 to 0.4 Hz, low frequency (lF), from 0.04 to 0.15 Hz, and very low frequency (vlF), from 0.0033 to 0.04 Hz, indexes of time-domain methods including full-course normal standard deviation of RR intervals (SDNN), standard deviation of the averages of NN intervals in all 5-min segments of the entire recording (SDANN), the square root of the mean of the squares of the successive differences between adjacent NNs (RMSSD), the proportion of NN50 divided by the total number of NNs (PNN50), and the total number of all NN intervals divided by the height of the histogram of all NN intervals measured on a discrete scale with bins of 7.8125 ms (triangular index). All HRV indexes were calculated by using BiovisualabHRV software (Biovisualab, Shanghai, China), which was modified from the original method described by Bauer *et al.*^14^.

### Echocardiography

An echocardiographic examination was performed using a Sonos 5500 type ultrasound machine (Philips, Best, the Netherlands) with a 2.5-Hz transducer. Measurement of the left ventricular ejection fraction (LVEF, normal value: >50%) was performed using Simpson’s biplane method. The measured parameters using the M-mode technique included end-systolic diameter (LVESd, normal range: 20–40 mm), left ventricular end-diastolic (LVEDd, normal range 35–56 mm), and left atrial diameter (LAd, normal range 27–40 mm).

All of the above measurements and the echocardiographic examinations for each patient were performed and analyzed by experienced technicians who were blinded to the clinical data and experimental design. Any uncertainties regarding results were resolved by discussion among senior technicians of the Department of Echocardiography and Department of Electrocardiograms.

### Electrocardiography

Resting 12-lead surface ECG was obtained in the supine position. For greater accuracy, measurements were performed with calipers and a magnifying lens. The Q-, R-, and S-wave complex (QRS) duration was calculated using the first to last sharp vector crossing the isoelectric line in leads V3–V6. Three continuous QRS duration values were detected, and their average value was defined as QRS duration. QT interval durations were recorded for three consecutive beats through leads II and V4, each QT interval was measured from the beginning of the QRS complex to the visual return of the T wave to the isoelectric line. When the T wave was interrupted by the U wave, the end of the T wave was defined as the nadir between the T and the U waves. Heart rate corrected QT interval (QTc) was performed by the Bazett formula, and QTc interval duration was defined as the mean duration of all QTc intervals measured. ST amplitude (ST J-point amplitudes) was measured in standard 12-lead ECG and also in special monitoring leads with right arm and left arm electrodes placed in subclavicular fossae and all chest lead electrodes placed at the level of V1 and V2 positions. Presences of atrial fibrillation and/or ventricular tachycardia were screened by the 24-h ambulatory electrocardiogram monitoring. Subject with one or more runs of nonsustained ventricular tachycardia of at least three beats in duration were recorded as positive for ventricular tachycardia. Electrocardiography was performed and analyzed by the same experienced technician, the final electrocardiographic result for each patient was reviewed by the chief technician.

### Statistical analysis

The distribution characteristics of all data were assessed first. Normally distributed data are presented as means ± standard deviation (SD), and skewed data are presented as medians (interquartile range). Paired or non-paired Student’s *t*-tests were used for comparisons within groups according to the data distribution characteristics; all were performed as two-sided tests. A *P* value <0.05 was deemed to indicate statistical significance. Statistical analyses were performed using SPSS software (ver. 17.0; SPSS Inc., Chicago, IL, USA). To avoid the influence among variables as much as possible, correlation patterns for each variable were analyzed quantitatively using Spearman’s rank correlation coefficient. Confounding factors and collinearity were examined by Spearman’s rank correlation coefficient array. To evaluate the performance of deceleration capacity, acceleration capacity and traditional HRV indexes in discriminating HF, HF patients and healthy controls were pooled, the area under the receiver operating characteristic curve (AUC) analysis was performed. Any differences in the AUC of indexes were examined by non-parametric tests of paired samples through bootstrapping with replicates of 10,000; the Bonferroni method was used to adjust the significance level for multiple comparisons. A multiple unconditional logistic regression model was used for our primary analysis of the independent effects of each variable. Potential associated factors for HF were selected according to univariate analyses (*P* < 0.1), Spearman’s rank correlation coefficient, and the principle of indexes. To determine the independent factors associated with HF, selected indexes were included in the binary multiple logistic regression analysis, in which HF was the dependent variable. The cut-off values for deceleration capacity and acceleration capacity in males and females were identified by searching the maximum log-rank statistics in ROC analysis.

## Results

### Characteristics of clinical testing indexes

In total, 130 patients with HF and 212 controls were enrolled in this study; the proportions of males in the HF and control groups were 68.5% and 19.3%, respectively. To avoid sex bias and observe any sex differences in the following analyses, HF patients and controls were analyzed by sex. The demographic, echocardiographic and electrocardiographic data, treatment, heart rate variability, acceleration capacity and deceleration capacity indexes were compared and are summarized in Table 1. In the males, age, LAd, LVEDd, LVESd, QRS duration, QTc interval, ST amplitude, percentages with premature atrial contraction, atrial fibrillation, premature ventricular contraction and ventricular tachycardia, average heart rate, slowest heart rate, RMSSD, PNN50, and acceleration capacity were significantly higher in the HF patients than controls (Table 1). Conversely, the LVEF, fastest heart rate, SDNN, hF, lF, vlF, triangle index, and deceleration capacity were significantly lower in the HF patients than controls (Table 1). In females, age, LAD, LVEDd, LVESd, QRS duration, QTc interval, ST amplitude, percentages with premature atrial contraction, atrial fibrillation, premature ventricular contraction and ventricular tachycardia, slowest heart rate, RMSSD, PNN50, and acceleration capacity were also significantly higher in the HF patients than controls (Table 1). Conversely, the LVEF, fastest heart rate, SDNN, vlF, triangle index, and deceleration capacity were also significantly lower in the HF patients than controls (Table 1). The distribution characteristics of all clinical testing indexes were similar between HF patients and controls across males and females, except average heart rate, hF, and lF, which lost significance in females. Of HF patients most patients were treated with ACE inhibitors and beta-blockers. In summary, our data showed the typical pathophysiological changes of systolic HF, including the left ventricular enlargement, left ventricular end-systolic diameter increase and LVEF <40%^1^. The deceleration capacity and absolute value of the acceleration capacity decreased significantly in both female and male HF patients.

### Correlation pattern among clinical testing indexes

Echocardiographic and cardiac electrophysiological indexes are highly correlated mutually. Although the Task Force of the European Society of Cardiology and the North American Society of Pacing Electrophysiology have recommended time and frequency domain measures of HRV^12^, these indexes are still highly correlated in principle. To observe the role of the acceleration and deceleration capacities of the heart in association with HF, the method used to handle the confounding factors is essential for the following analysis. To achieve this, the correlations among all variables were analyzed quantitatively using Spearman’s rank correlation coefficient. These indexes displayed complex correlations between each other and showed slight gender differences. As shown in Table 2, acceleration capacity was highly correlated with deceleration capacity, LVESd, SDNN, SDANN, lF and vlF, with absolute rho values more than 0.5. Deceleration capacity was highly correlated with LVEDd, LVESd, LVEF and vlF with rho values of −0.59, −0.63, −0.64 and 0.54, respectively (Table 2). Taken together, both acceleration capacity and deceleration capacity were significantly correlated with LAd, LVEDd, LVESd, LVEF, QRS duration, QTc interval, ST amplitude, slowest heart rate, fastest heart rate, SDNN, SDANN, lF, vlF and triangle index, while their correlation models are opposite (positive/negative), which might reflect the modulating effects of the parasympathetic and sympathetic nervous systems on the heart. Considering the principle, representativeness, avoiding multicollinearity and the goal of our study, triangle index, fastest heart rate, RMSSD, PNN50, and SDNN were selected to represent cardiac electrophysiological indexes to perform the following analysis, and acceleration capacity, deceleration capacity, and LVEF were also included.

### Risk factors associated with HF in multiple logistic regression

The analysis above showed the correlation pattern among clinical testing indexes. We next sought to determine the independence of these indexes using multiple logistic regression models. In the processes mentioned above, triangle index, fastest heart rate, RMSSD, PNN50, SDNN, and LVEF were selected to represent echocardiographic and cardiac electrophysiological indexes. To evaluate whether acceleration capacity and deceleration capacity were associated with HF independently after adjusting for confounders, in the following multivariate logistic regression analysis, acceleration capacity or deceleration capacity were used in a multiple logistic regression analysis together with triangle index, fastest heart rate, RMSSD, PNN50, SDNN, and LVEF. As shown in Table 3, in the models that included acceleration capacity, acceleration capacity, LVEF, SDNN, RMSSD, and PNN50 were independent risk factors for HF in males, while acceleration capacity, LVEF, and PNN50 were independent risk factors for HF in females. In the models that included deceleration capacity, deceleration capacity, LVEF, and RMSSD were independent risk factors for HF in males, while deceleration capacity (*P* = 0.053) and LVEF were independent risk factors for HF in females (Table 3).

### Performance of a single selected index in classifying HF

**Figure 1:**
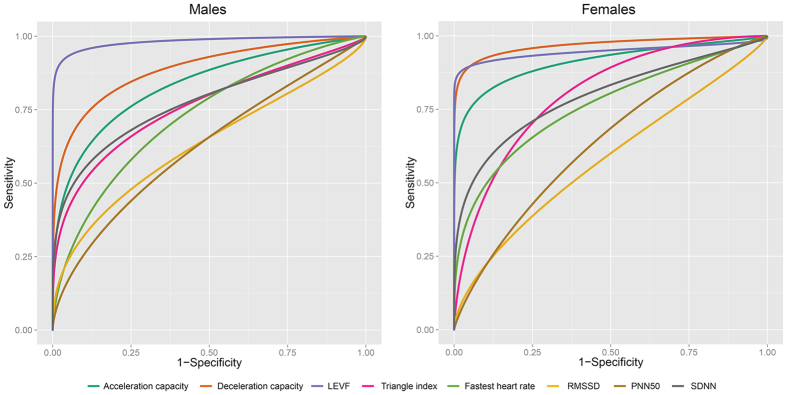
Performance of a single selected index in classifying HF. The performance of discrimination for each index was evaluated by receiver operating characteristic (ROC) analyses; areas under the ROC curve (AUC) and 95% confidence intervals are given for each ROC analysis and are shown in Table 4. For abbreviations, see Table 1.

To evaluate the performance of the triangle index, fastest heart rate, RMSSD, PNN50, SDNN, LEVF, acceleration capacity, and deceleration capacity in discriminate HF, a ROC curve was drawn for each variable. As shown in Fig. 1, in males, the three largest AUCs were for LVEF, 0.98 (0.96–1.00), acceleration capacity, 0.84 (0.78–0.89), and deceleration capacity, 0.88 (0.84–0.93) (Fig. 1, Table 4). Similarly, in females, LVEF, acceleration capacity, and deceleration capacity are also the three indexes with the highest AUCs: 0.92 (0.86–0.99), 0.97 (0.94–1.00), and 0.95 (0.88–1.00), respectively (Fig. 1, Table 4). Because all of these indexes showed some degree of performance in classifying HF, we wanted to determine the position of acceleration capacity and deceleration capacity in all indexes evaluated. To achieve this, differences in the AUC of all indexes were examined using non-parametric tests of paired samples through bootstrapping with 10,000 replicates. The Bonferroni method was used to adjust the significance level for multiple comparisons. As shown in Table 4, in males, the AUC for acceleration capacity was significantly lower than that for LVEF but significantly higher than those for RMSSD and PNN50, whereas deceleration capacity was the second best factor to classify HF in males (the best cardiac electrophysiological index). In females, the AUC for acceleration capacity was significantly higher than those for RMSSD and PNN50, the performance of LVEF was not significantly higher than that of acceleration capacity, and deceleration capacity was the best factor to classify HF in females.

### Cut-off values for acceleration capacity and deceleration capacity in classifying HF

The data above showed that acceleration and deceleration capacities possessed high performance in classifying HF. We next identified the cut-off values of deceleration and acceleration capacities by searching for the maximum log rank statistics. As shown in Table 5, the cut-off values for deceleration capacity and acceleration capacity in males were 4.55 (with 98.0% specificity and 67.4% sensitivity) and −6.15 (with 81.3% specificity and 75.6% sensitivity), respectively. The cut off values for deceleration capacity and acceleration capacity in females were 4.85 (with 95.3% specificity and 90.0% sensitivity) and −5.75 (with 86.0% specificity and 87.9% sensitivity), respectively.

### Beta-blockers and angiotensin-converting-enzyme (ACE) inhibitors treatments had not changed the HRV significantly in control population

In principle, Beta-adrenergic blockade therapy could inhibit sympathetic nervous activity and ACE inhibitors might effect on autonomic nervous modulation. In our study, most of patients were treated with beta-blockers and ACE inhibitors, to evaluate any possible influence of ACE inhibitors and beta-blockers administration on HRV, we pooled the male and female controls together and then divided them into treatment (n = 42) and non-treatment groups (n = 170). The HRV indexes of these two groups were further compared. The acceleration capacities in treatment group and non-treatment group were −7.7 (−9.1, −6.5) and −7.6 (−8.6 ~ −5.7), respectively; the deceleration capacities in treatment group and non-treatment group were 7.3 (6.1 ~ 8.3) and 7.2 (6.2 ~ 8.4), respectively, no significant difference was observed (Table 6). Of the traditional HRV indexes, no significant difference in any of the time domain or spectral HRV indices was observed either (Table 6). Although our evaluation was apart from the expectation, our result was consistent with a randomized double-blind parallel group-controlled trial study^17^. In this report, most of HF patients were treated by 23.75–47.5 mg metoprolol sustained-release tablets once daily, and for some of the patients with HF grade III–IV, the dose was reduced to 12.5 mg every day, these dosages were lower than Sanderson JE used, in his study, the dosage of metoprolol was 50 mg twice daily, even so, they had not observed any significant changes caused by metoprolol in the time domain or spectral HRV indices^17^.

For the effect of ACE inhibitors on autonomic nervous system, conflicting findings were reported. Sustained augmentation of parasympathetic tone and improvement of HRV were observed in HF patients after ACE inhibitor administration^18^,^19^, on the contrary, other researcher showed no effect of ACE inhibitor administration on HRV in HF patients^20^. In our study, most individuals were treated with beta-blockers and ACE inhibitors simultaneously. Although we could not distinguish the effect of each drug on the HRV, generally, our results suggested that administration of ACE inhibitors and beta-blockers had less influence on HRV.

## Discussion

The cardiac function of the heart is modulated opposingly by the sympathetic and parasympathetic nervous systems^7^,^21^. Disorders in the autonomic nervous system have been found to be associated with heart diseases^2^,^7^,^21^,^22^. However, with current biomedical technologies, it is difficult to quantify the regulation and pathological activity of the autonomic nervous system in the heart. For this reason, scientists have attempted to identify biomarkers that monitor autonomic nervous activity. Heart rate is a basic physiological marker that can reflect autonomic nervous activity and is apparently easy to measure; thus, variability in heart rate has been of interest in autonomic nervous activity-related studies on heart diseases^12^,^22^. Low HRV was found to be a predictor of sudden arrhythmic death; furthermore, HRV had been shown to be impaired in patients with HF^7^,^12^,^21^,^22^. However, the multitude of different measures of HRV are too complex in terms of demonstrating significance and meaning, and incorrect conclusions and excessive extrapolations have emerged^12^. Since the standardization of HRV measurements, research on HRV and autonomic nerves has improved. At the same time, novel approaches to interpret the role of the autonomic nervous system in the heart are continually being identified and proposed. Deceleration capacity and acceleration capacity are further examples^14^,^15^. In this report, we found deceleration capacity and acceleration capacity to be independent risk factors associated with HF both in males and females, although the p value for the OR for deceleration capacity was 0.053 in females. We believe this may be attributed to the small sample number of female HF patients. Second, in males, deceleration capacity was the best cardiac electrophysiological index in classifying HF, with an AUC of 0.88, and its performance was ranked only second to LEVF. Acceleration capacity was also a discrimination factor of HF, with an AUC of 0.84, and its performance was significantly higher than those of PNN50 and SDNN. In females, deceleration capacity was the best discrimination factor of HF with an AUC of 0.97; the performance of acceleration capacity was equal to that of LEVF (0.92 vs. 0.95). Third, we calculated the cut-off values for deceleration capacity and acceleration capacity for HF discrimination with high specificities and sensitivities. Given that deceleration capacity and acceleration capacity may represent the parasympathetic and sympathetic nervous system activities in the heart, our study suggests their roles in HF.

In HF, it has been recognized that the sympathetic nervous system is activated and that an imbalance between vagal and sympathetic activities occurs^23^. Clinical evidence has shown that in HF, afferent inputs from the arterial chemoreceptors, muscle metaboreceptors, and cardiopulmonary baroreceptors are activated, afferent inputs from arterial baroreceptors, pulmonary receptors, and ventricular mechanoreceptors are inhibited, and central excitatory mechanisms are activated^23^,^24^. Animal models of HF have also shown that sympathoexcitation and abnormal cardiovascular reflex function contribute to the activation of the sympathetic nervous system in HF^25^,^26^,^27^. As mentioned in the Introduction, in comparison with the sympathetic nervous system, we know little about the role of parasympathetic nervous activity in HF. In this report, deceleration capacity was demonstrated to be an independent risk factor for HF. Deceleration capacity was the best cardiac electrophysiological index in males; its performance in classifying HF was only slightly lower than that of LVEF. In females, deceleration capacity was the best HF-classifying index, and its performance was even higher than that for LVEF. These data suggest that vagal activity participates widely and extensively in the pathophysiological process of HF. Although our data do not provide any potential mechanisms regarding the role of vagal activity in HF, our data showed that deceleration capacity is significantly lower in HF patients, with cut-off values for HF diagnosis of 4.55 ms for males and 4.85 ms for females. As an indicator of sympathetic activity, acceleration capacity was an independent risk factor for HF, and its absolute value was also significantly lower in HF patients. In males, its performance in classifying HF was significantly lower than that of LVEF but significantly higher than those of RMSSD and PNN50. In females, the performance of acceleration capacity was equal to that of LVEF. Our data showed that increased parasympathetic activity, but not increased sympathetic activity, is dominant in HF.

Deceleration and acceleration capacities are new algorithm for HRV, which was based on 24-h ambulatory electrocardiogram monitoring^14^. The integrity and continuity of the calculation for deceleration and acceleration capacities ensured its application in clinical practice. Impaired heart rate deceleration capacity was demonstrated to be more powerful than LVEF and the conventional measures of HRV in predicting the mortality after myocardial infarction^14^. Decreased deceleration capacity was also found to be an independent predictor for sudden cardiac death in HF patients with systolic ventricular dysfunction^28^. Improves deceleration and acceleration capacity were observed in patients who underwent the cardiac rehabilitation program with controlled physical training^29^. Further more, compared to healthy individuals, reduced deceleration and acceleration capacities were observed in patients with type 1 diabetes^30^. Despite these encouraging results, more studies on the clinical value of deceleration and acceleration capacities are needed, e.g. no study compared any physiological and pathological significance of the deceleration and acceleration capacities in awake and sleep states; and the representativeness and comprehensiveness of HRV measures performed within 5 min period (recommended by the Task Force of the European Society of Cardiology and the North American Society of Pacing and Electrophysiology^12^) needs further assessment.

Echocardiographic indexes are independent of cardiac electrophysiological indexes in principle, while cardiac structural changes should influence cardiac electrophysiological changes^31^. Our correlation analysis showed that echocardiographic indexes and cardiac electrophysiological indexes displayed separability (Table 2), which might suggest that LAd, LVEDd, LVESd, and LVEF represent cardiac structural changes but do not reflect the relationship between cardiac structure and cardiac electrophysiology.

Limitations of this study included 1) the relatively small sample size for female HF patients, which might weaken the accuracy of the conclusions; and 2) since the autonomic modulation varies according to age, in this report, the age of patients with HF was significantly older than the age of controls, thus, we could not eliminate any possible impact of age difference on their cardiac electrophysiology. In this report, our goal is mainly focused on the deceleration and acceleration capacities of heart rate associated with HF, the associations between arrhythmia profile and HF were studied extensively^1^,^32^. HRV indexes included in this study were for control purposes. We had not included parameters such as QRS duration, QTc interval, ST amplitude, arrhythmias atrial fibrillation and ventricular tachycardia in subsequent analysis due to sample size limitation.

In conclusion, deceleration capacity and acceleration capacity are independent risk factors for HF; deceleration capacity was the best cardiac electrophysiological index to classify HF in males; deceleration capacity was the best HF-classifying index in females. Our study positions the roles of parasympathetic and sympathetic activity in HF and provides new insights into how parasympathetic and sympathetic activation affect HF.

## Additional Information

**How to cite this article**: Hu, W. *et al.* Deceleration and acceleration capacities of heart rate associated with heart failure with high discriminating performance. *Sci. Rep.* **6**, 23617; doi: 10.1038/srep23617 (2016).

## Figures and Tables

**Table 1:** Summary of clinical features of HF patients and controls.

	Male (n = 200)			Female (n = 142)		
Indexes	HF (n = 89)	Control (n = 111)	*P*	HF (n = 41)	Control (n = 101)	*P*
Demographic data						
Age (yrs)	65.5 (54.0 ~ 73.3)	59.0 (50.0 ~ 65.3)	<0.001	71.0 (58.0 ~ 78.5)	59.0 (50.0 ~ 67.0)	<0.001
Treatment						
ACE inhibitors	87 (97.8%)	21 (18.9%)	<0.001	40 (97.6%)	22 (21.8%)	<0.001
Beta-blockers	85 (95.5%)	23 (20.7%)	<0.001	38 (92.7%)	19 (18.8%)	<0.001
Echocardiography						
LAd (mm)	48.4 ± 7.7	37.2 ± 4.5	<0.001	47.0 ± 6.8	36.2 ± 4.9	<0.001
LVEDd (mm)	66.6 ± 10.2	49.2 ± 3.3	<0.001	65.2 ± 9.7	47.2 ± 3.8	<0.001
LVESd (mm)	57.0 (48.0 ~ 64.0)	31.0 (28.0 ~ 33.0)	<0.001	58.0 (45.5 ~ 63.5)	29.0 (27.0 ~ 31.0)	<0.001
LVEF (%)	32.0 (25.8 ~ 40.0)	68.0 (63.0 ~ 72.0)	<0.001	29.0 (25.0 ~ 45.0)	68.0 (64.0 ~ 73.0)	<0.001
Electrocardiogram						
QRS duration (ms)	118.2 (96.2 ~ 124.1)	88.2 (84.1 ~ 95.3)	<0.001	119.1 (96.7 ~ 125.7)	81.3 (78.1 ~ 89.1)	<0.001
QTc interval (ms)	429.3 (388.5 ~ 466.7)	388.3 (394.2 ~ 431.4)	<0.001	438.3 (392.1 ~ 479.1)	415.1 (395.7 ~ 441.2)	<0.001
ST amplitude (mV)	99.3 (71.2 ~ 110.1)	73.2 (64.1 ~ 82.3)	<0.001	53.2 (42.1 ~ 78.4)	30.2 (26.5 ~ 36.4)	<0.001
Arrhythmias						
Premature atrial contraction	20 (22.5%)	5 (4.5%)	<0.001	10 (24.3%)	4 (3.9%)	<0.001
Atrial fibrillation	18 (20.2%)	1 (0.9%)	<0.001	8 (19.5%)	2 (2.0%)	<0.001
Premature ventricular contraction	17 (19.1%)	1 (0.9%)	<0.001	8 (19.5%)	1 (1.0%)	<0.001
Ventricular tachycardia	8 (8.9%)	1 (0.9%)	0.012	4 (9.8%)	0 (0%)	0.006
Heart rate variability						
Average heart rate (bpm)	75.0 (64.8 ~ 84.0)	71.0 (66.0 ~ 77.3)	0.023	68.0 (61.5 ~ 81.0)	73.0 (66.0 ~ 76.0)	0.251
Slowest heart rate (bpm)	55.0 (49.0 ~ 66.0)	52.0 (47.8 ~ 56.3)	0.046	55.0 (48.5 ~ 63.5)	52.0 (49.0 ~ 56.0)	0.039
Fastest heart rate (bpm)	104.0 (91.5 ~ 113.0)	116.5 (107.0 ~ 127.3)	<0.001	99.0 (88.0 ~ 111.0)	118.0 (107.0 ~ 129.0)	<0.001
SDNN (ms)	78.0 (56.8 ~ 106.3)	116.5 (96.8 ~ 142.3)	<0.001	82.0 (61.5 ~ 92.5)	115.0 (97.0 ~ 135.0)	<0.001
SDANN (ms)	41.0 (25.0 ~ 61.0)	48.0 (39.8 ~ 59.0)	0.355	40.0 (25.5 ~ 47.0)	45.0 (37.0 ~ 53.0)	0.175
RMSSD (ms)	30.0 (21.3 ~ 45.3)	23.0 (18.0 ~ 29.3)	<0.001	29.0 (19.5 ~ 40.0)	24.0 (19.0 ~ 29.0)	0.035
PNN50 (%)	6.0 (3.0 ~ 14.8)	4.0 (2.0 ~ 7.3)	<0.001	7.0 (2.0 ~ 14.5)	4.0 (1.0 ~ 8.0)	0.024
hF (ms2)	106.5 (36.5 ~ 286.0)	126.2 (77.1 ~ 201.2)	0.017	98.5 (47.7 ~ 189.0)	122.2 (85.1 ~ 197.8)	0.219
lF (ms2)	105.3 (40.0 ~ 293.3)	324.0 (199.9 ~ 501.4)	0.022	125.2 (54.5 ~ 203.4)	264.1 (175.0 ~ 389.5)	0.052
vlF (ms2)	562.8 (225.9 ~ 1041.5)	959.3 (747.9 ~ 1282.8)	0.004	405.0 (224.9 ~ 877.5)	835.3 (660.1 ~ 1044.5)	<0.001
Triangle index	16.0 (12.0 ~ 24.0)	26.0 (19.8 ~ 30.0)	<0.001	17.0 (12.5 ~ 21.5)	24.0 (19.0 ~ 30.0)	<0.001
Deceleration and acceleration capacity						
Acceleration capacity (ms)	−4.5 (−6.2 ~ −3.4)	−7.7 (−9.3 ~ −6.4)	<0.001	−4.1 (−5.1 ~ −3.5)	−7.6 (−8.7 ~ −6.4)	<0.001
Deceleration capacity (ms)	3.9 (2.8 ~ 5.7)	7.2 (6.5 ~ 8.7)	<0.001	3.6 (3.1 ~ 4.3)	7.2 (6.1 ~ 8.1)	<0.001

Normally distributed data are presented as means ± standard deviation (SD), skewed data are presented as medians (interquartile ranges). Differences between HF and control groups were examined using the Kruskal-Wallis H test, Student’s t test, the chi-square and Fisher tests according to the characteristics of the data distribution. Abbreviations: HF, heart failure; ACE, angiotensin-converting enzyme; LAd, left atrial diameter; LVEDd, left ventricular end-diastolic diameter; LVESd, left ventricular end-systolic diameter; LVEF, left ventricular ejection fraction; QRS, QRS waves complex; QTc, corrected QT interval; ST amplitude, ST J-point amplitude; bpm, beat per minutes; SDNN, standard deviation of NN intervals; SDANN, standard deviation of the averages of NN intervals in all 5-min segments of the entire recording; RMSSD, root mean square of successive differences; PNN50, the mean number of times in full-course in which the change in successive normal sinus intervals exceeds 50 ms; hF, high frequency; lF, low frequency; vlF, very low frequency.

**Table 2:** Correlation coefficient array of clinical testing indexes.

	Acceleration capacity	Deceleration capacity	LAd	LVEDd	LVESd	LVEF	QRS duration	QTc interval	ST amplitude	Average heart rate	Slowest heart rate	Fastest heart rate	SDNN	SDANN	RMSSD	PNN50	hF	lF	vlF	Triangle index
Acceleration capacity	1.00																			
Deceleration capacity	−0.82**	1.00																		
LAd	0.33**	−0.47**	1.00																	
LVEDd	0.49**	−0.59**	0.70**	1.00																
LVESd	0.52**	−0.63**	0.69**	0.96**	1.00															
LVEF	−0.49**	0.64**	−0.63**	−0.86**	−0.95**	1.00														
QRS duration	−0.23*	0.32**	0.31**	0.43**	0.32*	−23*	1.00													
QTc interval	−0.31**	0.21*	0.21*	0.22*	0.18*	−33**	0.58*	1.00												
ST amplitude	0.13*	0.22*	0.12**	0.31**	0.31**	0.30	0.34**	0.34*	1.00											
Average heart rate	0.12	0.07	−0.15	−0.17*	−0.16	0.13	−0.23*	−0.34	0.34*	1.00										
Slowest heart rate	0.35**	−0.30**	−0.01	0.13	0.15	−0.15	0.34	0.32*	0.32**	0.69**	1.00									
Fastest heart rate	−0.34**	0.34**	−0.22*	−0.41**	−0.41**	0.40**	−0.23**	−0.34**	0.34	0.60**	0.12	1.00								
SDNN	−0.51**	0.39**	−0.19*	−0.34**	−0.38**	0.38**	−0.13**	−0.51**	0.23**	−0.23**	−0.56**	0.38**	1.00							
SDANN	−0.63**	0.23**	0.09	−0.07	−0.06	0.02	−0.14**	−0.35**	0.22	−0.32**	−0.44**	0.16	0.62**	1.00						
RMSSD	−0.25**	−0.13	0.22**	0.22**	0.24**	−0.26**	−0.27*	−0.23**	−0.21*	−0.37**	−0.32**	−0.18*	0.29**	0.71**	1.00					
PNN50	−0.23**	−0.13	0.19*	0.21*	0.22**	−0.23**	−0.30**	−0.53**	−0.19*	−0.31**	−0.26**	−0.17*	0.24**	0.70**	0.93**	1.00				
hF	−0.44**	−0.10	0.19*	0.08	0.11	−0.15	−0.20	−0.23**	0.13*	−0.15	−0.22**	0.07	0.39**	0.83**	0.78**	0.78**	1.00			
lF	−0.71**	0.32**	0.00	−0.15	−0.15	0.13	−0.11	−0.23**	0.22**	−0.11	−0.32**	0.27**	0.57**	0.90**	0.61**	0.61**	0.79**	1.00		
vlF	−0.54**	0.54**	−0.08	−0.21*	−0.23**	0.24**	−0.31**	−0.36**	0.10	−0.43**	−0.54**	0.10	0.53**	0.57**	0.24**	0.27**	0.15	0.409**	1.00	
Triangle index	−0.44**	0.44**	−0.25**	−0.30**	−0.36**	0.38**	−0.23*	−0.22**	0.24*	−0.20*	−0.38**	0.20*	0.55**	0.31**	0.04	0.01	0.08	0.29**	0.40**	1.00

The correlations between echocardiographic indexes, electrocardiogram indexes, heart rate variability indexes, deceleration capacity, and acceleration capacity were quantified using Spearman’s rank correlation coefficient. **the correlation was significant at the 0.01 level (2 tailed); *the correlation was significant at the 0.05 level (2 tailed). For abbreviations, see Table 1.

**Table 3:** Selected variables associated with HF in multiple logistic regression analysis.

	Male		Female	
Indexes	OR (95% CI)	*P*	OR (95% CI)	*P*
Acceleration capacity model				
Acceleration capacity	5.94 (1.74–20.25)	0.004	8.58 (1.79–41.1)	0.007
LVEF	0.20 (0.00–0.27)	0.02	0.06 (0.01–0.38)	0.003
SDNN	0.95 (0.92–0.99)	0.011	/	/
RMSSD	1.21 (1.06–1.39)	0.005	/	/
PNN50	/	/	1.23 (1.03–1.47)	0.023
Deceleration capacity model				
Deceleration capacity	0.13 (0.03–0.56)	<0.001	0.01 (0.00–1.06)	0.053
LVEF	0.21 (0.00–0.22)	0.001	0.01 (0.00–0.93)	0.047
SDNN	0.96 (0.92–1.00)	0.055	/	/
RMSSD	1.20 (1.01–1.43)	0.036	/	/

OR, odds ratio, CI, 95% confidence interval; For more abbreviations, see Table 1. ORs for continuous variables = OR for an increase of 1 unit except LVEF for which OR is for an increase of 10 units.

**Table 4:** Performance comparison in HF discrimination.

Index		AUC (95% CI)	P*
Male			
Acceleration capacity 0.84 (0.78–0.89)	Deceleration capacity	0.88 (0.84–0.93)	0.0248
	LVEF	0.98 (0.96–1.00)	0.0000
	Triangle index	0.77 (0.70–0.84)	0.0530
	Fastest heart rate	0.74 (0.66–0.81)	0.0421
	RMSSD	0.64 (0.56–0.72)	0.0021
	PNN50	0.62 (0.54–0.70)	0.0006
	SDNN	0.78 (0.72–0.85)	0.0627
Deceleration capacity 0.88 (0.84–0.93)	LVEF	0.98 (0.96–1.00)	0.0003
	Triangle index	0.77 (0.70–0.84)	0.0006
	Fastest heart rate	0.74 (0.66–0.81)	0.0009
	RMSSD	0.64 (0.56–0.72)	0.0000
	PNN50	0.62 (0.54–0.70)	0.0000
	SDNN	0.78 (0.72–0.85)	0.0009
Female			
Acceleration capacity 0.92 (0.86–0.99)	Deceleration capacity	0.97 (0.94–1.00)	0.1102
	LVEF	0.95 (0.88–1.00)	0.6233
	Triangle index	0.80 (0.72–0.88)	0.0068
	Fastest heart rate	0.77 (0.67–0.87)	0.0059
	RMSSD	0.62 (0.49–0.75)	0.0002
	PNN50	0.63 (0.51–0.75)	0.0001
	SDNN	0.81 (0.71–0.91)	0.0113
Deceleration capacity 0.97 (0.94–1.00)	LVEF	0.95 (0.88–1.00)	0.4858
	Triangle index	0.80 (0.72–0.88)	0.0000
	Fastest heart rate	0.77 (0.67–0.87)	0.0003
	RMSSD	0.62 (0.49–0.75)	0.0000
	PNN50	0.63 (0.51–0.75)	0.0000
	SDNN	0.81 (0.71–0.91)	0.0010

*The Bonferroni method was used to adjust the significance level for multiple comparisons: P < 0.05/13 (=0.0038) is considered to indicate significance. For abbreviations, see Table 1.

**Table 5:** Cut-off values for HF discrimination.

	Male		Female	
Index	Acceleration capacity	Deceleration capacity	Acceleration capacity	Deceleration capacity
Cut-off	−6.15	4.55	−5.75	4.85
Specificity	0.813 (0.504–0.892)	0.980 (0.818–1.000)	0.860 (0.710–0.970)	0.953 (0.720–1.000)
Sensitivity	0.756 (0.597–0.841)	0.674 (0.414–0.769)	0.879 (0.542–0.963)	0.909 (0.778–0.909)

The unit of acceleration capacity and deceleration capacity is ms. Specificity and sensitivity are expressed as values and 95% confidence intervals.

**Table 6:** Medication had not changed the HRV significantly in control population.

Index	Treatment group (n = 42)	Non-treatment group (n = 170)	*P*
SDNN (ms)	117.0 (75.1 ~ 127.5)	113.0 (77.0 ~ 134.0)	0.0673
SDANN (ms)	47.1 (28.9 ~ 47.9)	46.2 (33.5 ~ 53.2)	0.4321
RMSSD (ms)	23.8 (21.1 ~ 41.0)	24.0 (18.7 ~ 27.1)	0.3234
PNN50 (%)	4.1 (2.1 ~ 7.4)	4.0 (1.5 ~ 8.1)	0.2545
hF (ms2)	123.2 (67.9 ~ 198.3)	119.5 (88.2 ~ 204.3)	0.4345
lF (ms2)	287.3 (175.3 ~ 423.1)	268.9 (179.0 ~ 391.2)	0.0765
vlF (ms2)	947.0 (724.9 ~ 1377.5)	897.9 (683.7 ~ 1214.3)	0.0698
Triangle index	27.0 (19.5 ~ 31.5)	25.1 (19.2 ~ 30.3)	0.2342
Acceleration capacity (ms)	−7.7 (−9.1, −6.5)	−7.6 (−8.6 ~ −5.7)	0.3454
Deceleration capacity (ms)	7.3 (6.1 ~ 8.3)	7.2 (6.2 ~ 8.4)	0.4522

Data are presented as medians (interquartile ranges). Differences were examined using the Mann-Whitney test. The dosages of beta-blocker treatment are 23.75–47.5 mg metoprolol sustained-release tablets once daily, and for some of the patients with HF grade III–IV, the dose was reduced to 12.5 mg every day. The dosages of ACE inhibitor (captopril) are 25–50 mg twice daily. For abbreviation, please see Table 1.
